# Assessment of the Self-Perception of Dental Appearance, Its Comparison with Orthodontist's Assessment and Demand for Treatment in Eastern Nepalese Patients

**DOI:** 10.1155/2014/547625

**Published:** 2014-08-13

**Authors:** Varun Pratap Singh, Amita Sharma, Deepak Kumar Roy

**Affiliations:** ^1^Department of Orthodontics, College of Dental Surgery, BP Koirala Institute of Health Sciences, Dharan 7053, Nepal; ^2^Department of Dentistry, SHKM Government Medical College, Mewat, Haryana, India; ^3^Department of Conservative Dentistry and Endodontics, College of Dental Surgery, BP Koirala Institute of Health Sciences, Dharan, Nepal

## Abstract

*Aims*. The aim of this study was to assess the self-perception of dental appearance among Eastern Nepalese patients using aesthetic component (AC) of the index of orthodontic treatment need (IOTN) and to compare it with that of an orthodontist's assessment using the same scale and determine whether gender, area of residence, and level of education influence subject's self-perception and orthodontist's ratings.* Methods*. A total of 252 subjects (equal number of male and female) were conveniently selected. The average ages of subjects were 22.33 ± 2.114 years. The level of subject's perception and orthodontist's assessment was analyzed by nonparametric Chi square test. Kappa coefficient was done to verify its agreement. The Spearman's correlation test was used to check the association of educational level and age. Mann-Whitney test was used to check the associations of sex and areas of residence.* Results*. The demand for treatment was significantly associated with the perception of the subject and orthodontist's assessment. However, age, gender, and educational level were statistically insignificant in influencing subject perception and orthodontist's assessment.* Conclusion*. Patient's self-perception should be given equal importance while planning orthodontic treatment.

## 1. Introduction

A person having an attractive smile is appreciated by everyone. People always want to know how they look and what others think about their physical appearance. Therefore, a pleasing smile and an attractive facial appearance help to improve one's self-esteem and have a positive impact on building social as well as professional relations. One with poor dental appearance may have a negative impact [1].

Enhancing dentofacial esthetics is one of the primary goals of orthodontic treatment. Frequently, people desire orthodontic treatment to address their esthetics concerns [2, 3]. A number of studies have shown that children have developed a self-perception for the need of orthodontic treatment [3–10]. Orthodontic treatment is determined mostly by the objective assessment. The patient's perception toward seeking a dental treatment is usually ignored [2, 11].

**Figure 1:**
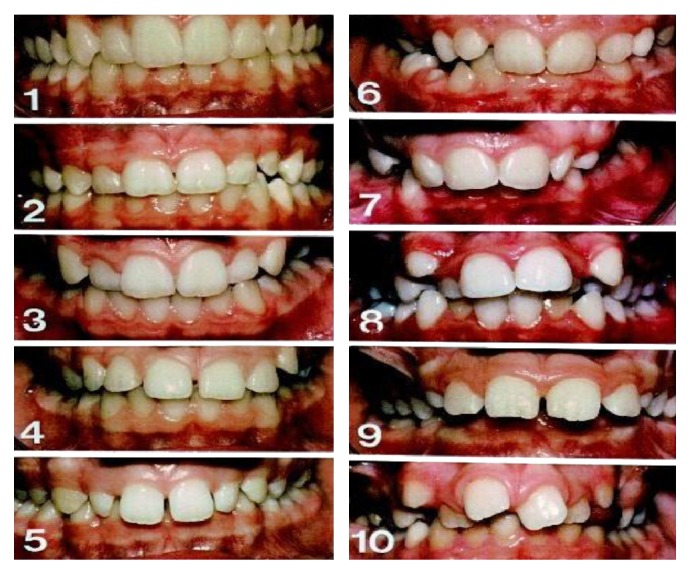
Aesthetic component scale.

The index of orthodontic treatment need (IOTN) has two components: one component relates to dental and functional health (dental health component (DHC)) and the other is based on aesthetic impairment of malocclusions (aesthetic component (AC)). The former is concerned only about dental health [12, 13] and the later deals with psychological need for orthodontic treatment [14]. The AC IOTN (Figure 1) consists of ten colorful photos of the occlusion of anterior teeth which is a standard method of assessing dental aesthetics. The data concerning the self-perception of malocclusion and the need of orthodontic treatment are available for many populations, [5–10, 15–20]; however, the perception of malocclusion in Nepalese population is being studied for the first time.

The objectives of this study were to

- assess the self-perception of dental appearance among Eastern Nepalese population using aesthetic component (AC) of the IOTN index,
- compare the perception of dental appearance of the subject with orthodontic assessment,
- determine if gender (male/female) and residence (rural/urban) influence subject's self-perception and orthodontist's rating.

## 2. Methods

A hospital based cross-sectional study was conducted among 252 patients aged 17–29 years. The sample was selected from patients visiting the Department of Orthodontics, College of Dental Surgery, BP Koirala Institute of Health Sciences, Dharan, Nepal, between January 2011 to January 2012. Convenience sampling was done and an equal number of males and females were selected to enable comparison.

The subjects for the study were allowed to assess their own occlusion using color photographs of the aesthetic component. A series of 10 colorful photographs showing the range of dental attractiveness, number 1 having the most and number 10 the least attractive, were put in front of the subject to judge their occlusions and were asked individually to rate their teeth by seeing the photograph. Along with it, the patients were asked about their level of education, number of schooling years, area of residence, and demand for orthodontic treatment. At the same time, the orthodontist rated the subject's occlusions using the AC scale. Finally, the ratings done by the patient and orthodontist were compared. To make the study more reliable a mirror and a lip retractor were provided to the patient. To make the test more valid, out of 252 subjects, 52 randomly selected subjects were reexamined 6 weeks after their initial examinations. After the ratings were noted, the grades for the treatment need were calculated. The AC has numbers for each photograph which signifies the grades for treatment need (Figure 1). The grades are as follows:

 AC Grade 1–4: no need for treatment,
 AC Grade 5–7: borderline/moderate need for orthodontic treatment,
 AC Grade 8–10: definite need for orthodontic treatment.

### 2.1. Statistical Procedures

A SPSS statistical package (Statistical Package for the Social Sciences Version 17.0) was used to analyze the data. Chi-square test was applied to test the perception of subject and the orthodontist. Kappa coefficient was done to verify its agreement. Spearman's correlation test was applied to see the association between subject's and orthodontist's perception on education level and age. Mann-Whitney test was applied to check the association between subject's perception and orthodontist's assessment with respect to areas of living (rural/urban) and gender (male/female).

## 3. Results

**Figure 2:**
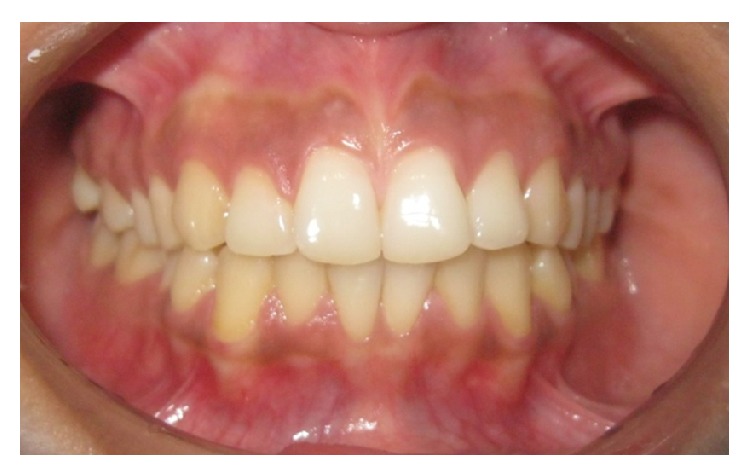
IOTN-AC Grade 1–4: no need for treatment.

**Figure 3:**
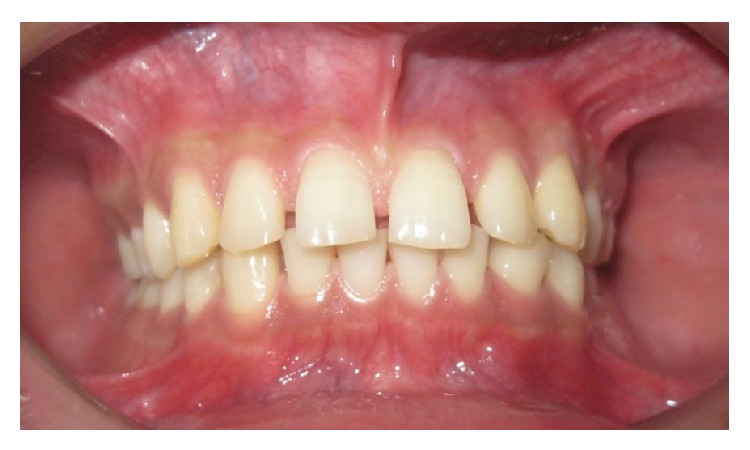
IOTN-AC Grade 5–7: borderline/moderate need for orthodontic treatment.

**Figure 4:**
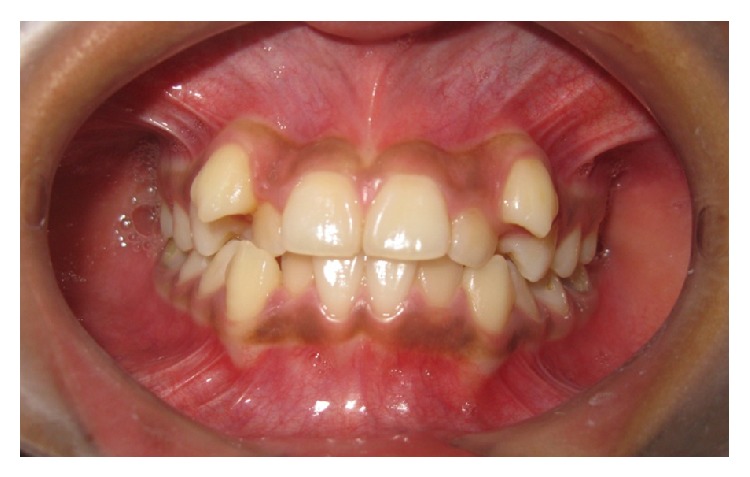
IOTN-AC Grade 8–10: definite need for orthodontic treatment.

A total of 252 subjects (equal number of male and female) took part in the study. The average age was 22.33 ± 2.114 years. The level of education was 13.83 ± 1.82 schooling years. The subjects consisted of 128 individuals from rural and 124 from urban areas. Treatment was demanded by 123 subjects (Table 1). Figures 2, 3, and 4 show IOTN-AC grading according to patients perception.

There were significant differences between subject's perception and orthodontist's rating (*P* = 0.000). The Kappa agreement was 48.7%. Most of the patients rated them in the “no need” category of IOTN-AC (215 patients) while the orthodontist rated (172 patients) under the same category. However, the orthodontist rated almost thrice the patients in “borderline need” (72 patients orthodontist, 25 patients self-perception) category as compared to the subjects (Table 2). The data were statistically insignificant with respect to age, sex, educational level, and place of residence. However, the demand of treatment was significantly related to the scores assigned by both the subjects and orthodontist (Tables 3 and 4).

## 4. Discussion

The results of this study indicated that the orthodontist's assessment was dissimilar with the subject's perception. More subjects perceive that they do not need orthodontic treatment as compared to orthodontist's assessment for “no need” group. The reason may be that layperson tends to have a less critical view of the same malocclusions assessed by the professionals as supported by Shaw et al. [14], Hunt et al. [21], Trivedi et al. [22], and Graber and Lucker [23]. For moderate need group, again there was disparity in orthodontist's assessment and subject perception with less subjects perceiving moderate need as compared to orthodontist. The reason may be the difficulty for the general population to differentiate between the features of malocclusions such as deep bite and increased overjet while seeing the photograph which is only understood by an expert and is rated in treatment need grade of AC. For definite need group the patients perceived them of having more severe malocclusion as compared to orthodontists and are consistent with Reichmuth et al. [24] who found that subjects rated themselves as having worse occlusion in publicly funded clinics. In this study, it appeared that the gender and age had no significant relation which is similar to the study done by Albarakati [1] in Saudi Arabia. A study done by Aikins et al. [25] to check the self-perception of malocclusion among Nigerian adolescents found that there was no significant relation with respect to gender but adolescents aged 16–18 had an increased level of perception of need. Further, in the same study, males were found to be more in need of treatment by orthodontist. Al-Balkhi and Al-Zahrani [26] also found lack of significant difference between both genders. On the contrary, Burden and Holmes [27] found that there were significantly more males than females who were in need for orthodontics treatment. The present study shows insignificant relation toward the level of education in relation to the perception of subject and the assessment of orthodontist. Unlike the study done by Bellot-Arcís et al. [28] in a Spanish adult population in which it was found that the level of education is statistically significant as the treatment need was more higher in secondary/higher education. The present study also depicts insignificant relation between the perception of subjects and orthodontist's assessment with areas of residence (rural/urban) which is supported by a study done among north Jordanian school children by Abu Alhaija et al. [10].

This study was conducted in a tertiary health care center in Eastern Nepal which has patients from a wide range of social backgrounds. The sample does not represent the whole population of this age group residing in Eastern region but gives an overview of the assessment of orthodontist and self-perception of subjects who took part in the study.

## 5. Conclusions

1. A significant difference was found between the orthodontist's assessment and perception of subjects regarding the attractiveness of occlusion in this hospital based Nepalese population of patients between ages 17 and 29 years.
2. Age, gender, level of education, and area of living were found to be statistically insignificant factors pertaining to perception of subjects and orthodontist's assessment.
3. For effective orthodontic care the perception of both subject and professional assessment must be taken into consideration.

## Figures and Tables

**Table 1:** Showing the descriptive statistics.

Sex		Age	Educational level	Rural/urban		Demand for treatment	
M	F	17 to 29 years	10 to 18 years	R	U	D	ND
126	126	Mean—22.33 ± 2.114	Mean—13.83 ± 1.828	128	124	123	129

R: rural; U: urban; D: demand; ND: no demand.

**Table 2:** Comparison of subject's perception and orthodontist's assessment of IOTN-AC.

IOTN-AC	Grades			Chi-square nonparametric	Kappa measurement of agreement
	No need (1 to 4)	Borderline need (5 to 7)	Definitive need (8 to 10)		
Subject's perception	215	25	12	215.32 Sig. 0.000	0.487
Orthodontist's assessment	172	72	08		

**Table 3:** Correlation between subject's perception and orthodontist's assessment with educational level and age.

Variables	Subject's perception		Orthodontist's assessment	
	CC	Sig. (2 tailed)	CC	Sig. (2 tailed)
Educational level	−0.062	0.325	−0.095	0.132

Age	0.062	0.328	0.024	0.702

CC: correlation coefficient.

**Table 4:** Effect of variables (rural/urban, sex, and demand) on subject's perception and orthodontist's assessment of IOTN-AC.

Asymp. sig (2 tailed)	Layman's perception	orthodontist's assessment
Rural/urban	0.510	0.726
Sex	0.875	0.412
Demand	0.000∗	0.000∗

^*^ *P* < .01.
